# Annual Report 2022

**DOI:** 10.3934/microbiol.2023007

**Published:** 2023-02-25

**Authors:** Xu Guo

**Affiliations:** Room 703, Luoke Times Center, Anli Road, Chaoyang District, Beijing 100101, China

## 1. Journal summary

AIMS Microbiology is an international Open Access journal devoted to publishing peer-reviewed, high quality, original papers in the field of microbiology. Together with the Editorial Office of AIMS Microbiology, I wish to testify my sincere gratitude to all authors, members of the editorial board and reviewers for their contribution to AIMS Microbiology in 2022.

In 2022, We received more than 200 manuscripts and 40 of them were accepted and published. These published papers include 23 research articles, 11 review articles, 2 editorials, 2 communications and 1 brief report papers. The authors of the manuscripts are from more than 20 countries. The data shows a significant increase of international collaborations on the research of microbiology.

An important part of our strategy has been preparation of special issues. 2 special issues published more than five papers. AIMS Microbiology have invited 17 experts to join our Editorial Board in 2022. We will continue to renew Editorial Board in 2022.

We hope that in 2023, AIMS Microbiology can receive and collect more excellent articles to be able to publish. The journal will dedicate to publishing high quality papers by regular issues as well as special issues organized by the members of the editorial board. We believe that all these efforts will increase the impact and citations of the papers published by AIMS Microbiology.

*On behalf of*

*AIMS Microbiology Editorial Board*

## 2. Editorial development

### 2.1. Manuscript statistics

The submissions of our AIMS Microbiology journal in 2022 increased. In 2022, AIMS Microbiology published 4 issues, a total of 40 articles were published online, and the category of published articles is as follows:

**Table:** 

Type	Number
Research	23
Review	11
Editorial	2
Communication	2
Brief report	1

**Figure:**
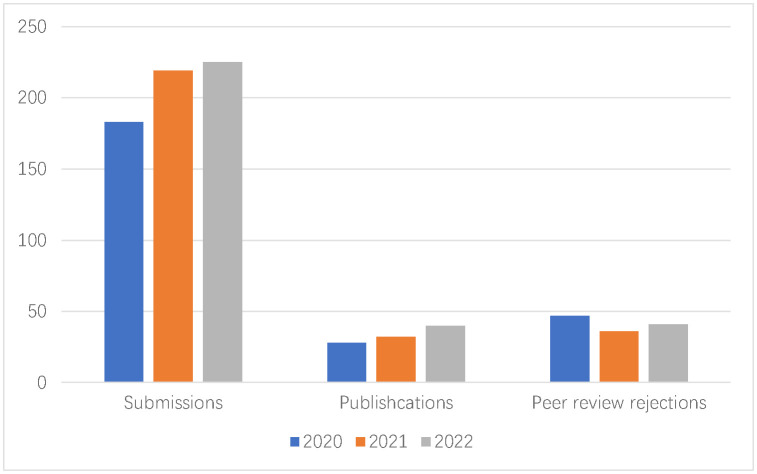


Peer Review Rejection rate: 49%

Publication time (from submission to online): 75 days

### 2.2. Some special issues with more than 5 papers

Organizing high-quality special issue is a very important work in 2022. In 2022, 7 special issues were called. Listed below are some examples of issues that have more than 5 papers. We encourage Editorial Board members to propose more potential topics, and to act as editors of special issues.

**Table:** 

Special issue	link	Papers
Biotechnological applications of microorganisms in Industry, Agriculture and Environment	https://www.aimspress.com/aimsmicro/article/6262/special-articles	8
Antimicrobials and Resistance	https://www.aimspress.com/aimsmicro/article/6209/special-articles	5

### 2.3. Editorial Board members

AIMS Microbiology has Editorial Board members representing researchers from 20 countries, which are shown below. We are constantly assembling the editorial board to be representative to a variety of disciplines across the field of microbiology. AIMS Microbiology has 81 members now, and 17 of them joined in 2022. We will continue to invite dedicated experts and researchers in order to renew the Editorial Board in 2022.

**Figure:**
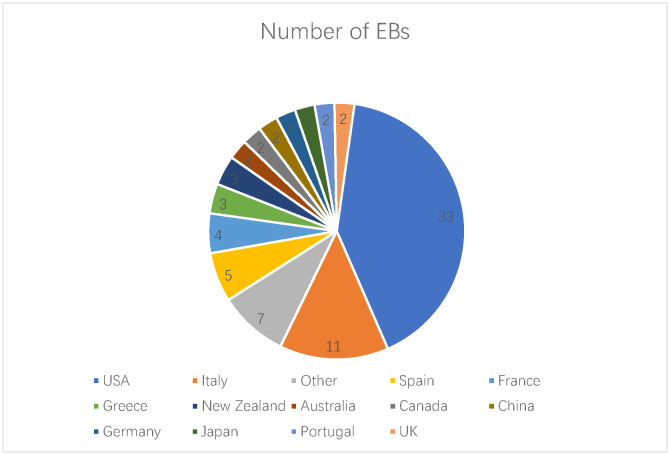


### 2.4. Articles metrics

Top 5 Cited Papers in last 2 years.

**Table:** 

No.	Article	Citations
1	Salmonella spp. quorum sensing: an overview from environmental persistence to host cell invasion	10
2	Ways to improve biocides for metalworking fluid	9
3	Yeasts in different types of cheese	7
4	Exploring endophytes for in vitro synthesis of bioactive compounds similar to metabolites produced in vivo by host plants	7
5	Listeria monocytogenes isolates from Western Cape, South Africa exhibit resistance to multiple antibiotics and contradicts certain global resistance patterns	6

### 2.5. Summary & plan

#### 2.5.1. Summary

In the recent two years, our journal has developed much faster than before; Our journal has been indexed in Web of Science, Scopus and PubMed databases. We received more than 200 manuscript submissions and published 40 papers in 2022. We have added 17 new Editorial Board members.

#### 2.5.2. Plan in 2023

In 2023, we expect to publish more articles to enhance the reputation. We will invite more experts in the field of microbiology to publish a review or research article. To set a goal, we would like to publish 40 high-quality articles in 2023. In 2023, we will continue to update our editorial board. We hope that more experts in the field of microbiology can help us review and guest special issues.

